# A Narrative Review of the Design of Ultrasound Indices for Detecting Enthesitis

**DOI:** 10.3390/diagnostics12020303

**Published:** 2022-01-25

**Authors:** Yeohan Song, Sheryl Mascarenhas

**Affiliations:** Department of Internal Medicine, Division of Rheumatology, The Ohio State University Wexner Medical Center, 543 Taylor Ave., Columbus, OH 43203, USA; Yeohan.Song@osumc.edu

**Keywords:** enthesitis, enthesopathy, ultrasound, score, index

## Abstract

With the increased utilization of musculoskeletal ultrasound in clinical practice, there has been rapid proliferation of publications on sonographic evaluation of enthesitis. This has led to the development of multiple new approaches to scoring sonographic findings in the detection of enthesitis, with variations including entheseal sites and sonographic features that limit cross-study comparisons. Furthermore, despite efforts to standardize the definition of enthesitis, there is still heterogeneity in the sonographic features included in existing ultrasound scores, and additional adjustments are required to distinguish active inflammatory changes from non-inflammatory conditions and to adjust for demographic features associated with increased prevalence of abnormal sonographic findings. This review provides an update on the current landscape of ultrasound scoring systems for enthesitis and emphasizes the importance of future data-based ultrasound scoring systems to improve the distinction between inflammatory and non-inflammatory or degenerative changes of the enthesis.

## 1. Introduction

Ultrasound has been increasingly utilized as an imaging modality to improve detection of inflammation of the enthesis, termed enthesitis, and has informed the understanding of associated inflammatory changes that extend to the adjacent fibrocartilage, bone, and, if present, bursa [1,2]. Detection of entheseal vascularization by power Doppler (PD) has also been demonstrated to have a role in identifying early cases of spondyloarthritis [3]. Furthermore, this imaging modality has also been shown to improve detection of enthesitis in patients with other chronic conditions that are not traditionally considered inflammatory, such as fibromyalgia, which can present with similar pain at entheseal sites and may at times be difficult to distinguish clinically [4,5]. In the aim of improving standardization of sonographic findings, the Outcome Measures in Rheumatology (OMERACT) Ultrasound Task Force published a consensus-based definition of enthesitis that included hypoechogenicity, increased thickness at the enthesis insertion, erosions, calcifications, enthesophytes, and PD signal of the enthesis within 2 mm from bony cortex in their final sonographic definition of enthesitis [6]. Ultrasound scoring systems or indices, defined as summative measures of sonographic abnormalities at specified entheseal sites, have previously been used as indicators of the sonographic detection of enthesitis [2,3,4]. However, not all of these characteristic features of enthesitis have been uniformly included in the available ultrasound scoring indices.

There have been different approaches to the categorization of sonographic findings of enthesitis, such as classifying calcifications and enthesophytes as a single category in the Madrid Sonographic Enthesitis Index (MASEI) versus separating these findings into different categories as in the more recently proposed preliminary enthesitis scoring system by the Group for Assessment of Psoriasis and Psoriatic Arthritis (GRAPPA) [7,8]. Additionally, there have been fundamental differences in the anatomic structures included when examining the enthesis with ultrasound. Benjamin and McGonagle first conceptualized the “enthesis organ” as comprised of the tendon or ligament insertion, fibrocartilage, bursa, fat pad, adjacent trabecular bone networks, and deeper fascia, such that bursitis was considered an integral component of enthesitis [9]. Based on the enthesis organ concept, the modified Glasgow Ultrasound Enthesitis Scoring System (GUESS) for enthesitis by Aydin et al. included bursitis [10]. However, both the OMERACT group and D’Agostino et al. identified bursae as separate structures and excluded bursitis from their sonographic definitions of enthesitis [6,11].

In addition to discrepancies in the anatomic structures included when evaluating enthesitis by ultrasound, there is no consensus on which entheseal sites to include as part of the diagnostic exam. A review on ultrasound assessment of enthesitis illustrated the heterogeneity of examined tendon insertions, sonographic features reported, and ultrasound score or index used that limits cross-study comparison [12]. Cross-study comparisons among published studies have been limited due to variability in the ultrasound scoring systems used, as several studies modified the scoring systems with variations in entheseal sites, sonographic features, and scoring scales [2,3,7,13]. For example, Balint et al. initially included five bilateral lower extremity entheses in the GUESS enthesitis scoring system, as did the subsequent Sonographic Entheseal Index (SEI) by Alcalde et al. and the modified GUESS by Aydin et al. [2,10,13]. However, D’Agostino et al. excluded the distal patellar ligament insertions and instead included the gluteus medius entheses along with two additional entheses of the bilateral upper extremities [3]. In the MASEI, de Miguel et al. included all of the original entheses from the GUESS while also adding the triceps entheses [2,7].

Despite the lack of a uniform approach to the ultrasound evaluation of entheses, moderate to strong inter-observer agreement in the use of ultrasound scoring systems has been demonstrated [6,14]. For example, the OMERACT group reported mean inter-observer agreement among a group of 11 rheumatologists with extensive ultrasound experience (>10 years) ranging from 70% for enthesophytes to 10% for entheseal thickening, with an overall inter-observer agreement for enthesitis of 60% (prevalence- and bias-adjusted kappa 0.6) [6]. Similarly, Milutinovic et al. reported excellent overall inter-observer reliability for the Belgrade Ultrasound Enthesitis Score (BUSES) (intra-class correlation coefficient of 0.990, 95% confidence interval 0.985, 0.993), though only two ultrasound operators were included in the study [14].

A consensus on how to assess enthesopathy by ultrasound is important in distinguishing inflammatory from non-inflammatory causes of entheseal pain, such as entheseal inflammation associated with psoriatic arthritis and spondyloarthritis versus degenerative changes due to repetitive mechanical injuries. By more appropriately distinguishing non-inflammatory causes, unnecessary immunosuppression and associated complications can also be avoided. Additionally, recent studies indicate that there remains a high prevalence of abnormal sonographic findings that may be misidentified as enthesitis among otherwise healthy volunteers, as well as among those with certain demographic features, such as male sex, higher BMI, or older age, underscoring the need for further improvements upon existing ultrasound scoring systems to more reliably distinguish enthesitis from non-inflammatory causes [15,16]. To this end, the term “enthesosis” has previously been introduced in recognition of non-inflammatory chronic degenerative changes at an enthesis as distinctly separate from enthesitis, which, based on the etymology and historical use of the suffix “-itis” can be used specifically to refer to inflammation of the enthesis [17].

In this review, we examine the ultrasound scoring systems for enthesitis published to date and recent findings on the prevalence of non-inflammatory sonographic entheseal abnormalities in order to inform the development of an ultrasound scoring system that improves the distinction of enthesitis from non-inflammatory enthesopathy.

## 2. Methods

**Figure 1:**
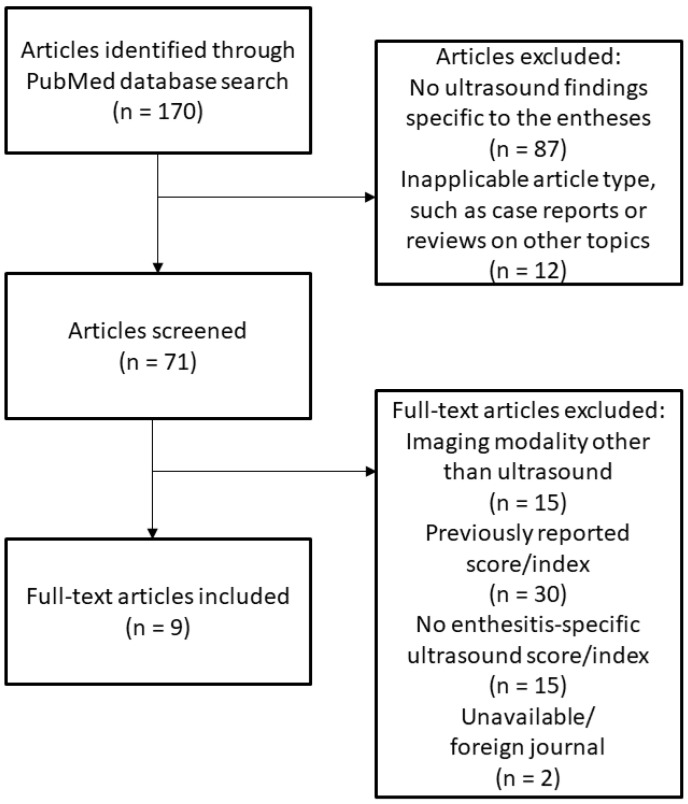
Literature Review Diagram.

A narrative literature review was conducted by performing a search of the PubMed databases using the terms “enthesitis”, “ultrasound”, “score”, and “index” using nested search terminology such that studies were identified with the terms “enthesitis” and “ultrasound” and either “score” or “index”. All available studies since 1994 that included the aforementioned search terms were included. This review was conducted by the first author (Y.S.) and included the time period between 1994 to 2021 (last accessed on 17 October 2021). The initial search resulted in 350 articles; the titles and abstracts of publications from 1994 to 2016 (180 articles) were reviewed, with findings confirming the previously identified ultrasound scoring systems in prior reviews [11,12]. The original full texts of these studies were then manually reviewed and analyzed for this review. An additional 170 publications were further screened, excluding studies without enthesitis-specific ultrasound findings and article types not relevant to this review, such as case reports. The resulting full-text articles were then manually reviewed for scoring systems used to evaluate enthesitis by ultrasound. The ultrasound scoring systems described in the resulting full-text articles were then analyzed in terms of the following: entheseal sites examined, sonographic features included, and scoring scales used (i.e., binary or weighted scores). Figure 1 illustrates the search methods used for this review. Once analyzed, these scoring systems were compared and contrasted to identify shared and differentiating characteristics. Articles identified during this review reporting abnormal sonographic findings of the entheses in the absence of enthesitis were used to inform potential modifications to the ultrasound scoring systems for enthesitis to further distinguish inflammatory from non-inflammatory causes of detected abnormalities.

## 3. Results

In addition to the previously published ultrasound scoring systems included in a previous review [11], an additional 170 publications were initially identified in this literature search, which were analyzed separately from and in addition to a previous systematic review of enthesitis scoring indices [12]. The flow diagram of this literature search is shown in Figure 1. After screening and manual review, a total of nine studies reporting unique ultrasound scoring systems or indices for enthesitis in addition to a published modification to a previously reported index (modified GUESS) were included in this review and collectively analyzed alongside the four major indices previously reported (GUESS, SEI, MASEI, and D’Agostino Scoring System) [11].

Of the 14 ultrasound scoring systems for enthesitis reported in the literature, the majority (71.4%) were published within the past decade. Of these more recent scoring systems, all included PD signal at tendon or ligament insertion as a factor in the sonographic assessment for enthesitis, while only 50% of those reported previously included this feature, instead relying primarily on grayscale or B mode findings.

Among the entheseal sites included, only lower extremity entheses were required in four (28.6%) scoring systems. The total number of examined entheseal sites ranged from a minimum of eight entheses to a maximum of 16 entheses of the upper and lower extremities, with a median of 12 examined entheses. The most commonly assessed entheseal site was the Achilles tendon, which was included in all of the published scoring systems. While the patellar ligament was included in all of the scoring systems, there were differences in whether the origin or the insertion was included, with most (71.4%) including both origin and insertion while three (21.4%) included only the origin of the patellar ligament at the patellar apex and one (7.1%) included only the insertion at the tibial tuberosity. The quadriceps tendon was the next most frequently assessed, included in 92.9% of the scoring systems. The plantar fascia was examined in 85.7% of the indices, followed by the common extensor tendon of the lateral epicondyle (57.1%), which was the most frequently assessed enthesis of the upper extremity. Interestingly, one study allowed for the inclusion of additional entheseal sites in ultrasound score evaluation alongside the “mandatory” entheses based on whether tenderness was elicited on clinical examination [18].

Of the sonographic features used to determine the presence of enthesitis, the most commonly included abnormalities were of increased thickness of the tendon/enthesis and the presence of enthesophytes or erosions, which were reported in all of the scoring systems. The majority of scoring systems also included detection of hypoechogenicity (85.7%) and identification of pathologic intra-tendinous calcifications (78.6%) as distinct from enthesophytes. However, only eight (57.1%) of the scoring systems included bursitis in the evaluation of enthesitis. As noted above, abnormal findings of increased signal on PD were included in all of the more recently (i.e., within the past decade) published ultrasound scoring systems for enthesitis.

Several ultrasound scoring systems utilized semi-quantitative or quantitative measurements to report abnormal findings [4,7,8,14]. Fewer than half of the scoring systems (42.9%) employed a binary (absent/present) score for detected abnormalities. Of the weighted systems used, however, there was no consensus on the approach used to attribute increased importance to specific findings. For example, in the preliminary GRAPPA system, PD abnormalities were weighted based on a semi-quantitative scale of 0–3, with 0 representing “absent”, 1 = “mild”, 2 = “moderate”, and 3 = “severe” Doppler signal intensity [8]. On the other hand, in the ULISSE study, a quantitative approach across a similar scale was used according to number of vessels detected (0 = no vessels, 1 = 1 to 3 vessels, 2 = 4 to 5 vessels, 3 = >5 vessels) [4]. Still others used a weight when PD signal abnormalities were detected at the entheses, but did not use a scale, with the weighted value differing across studies (e.g., 0 = absent, 3 = PD signal present for MASEI, or 0 = absent, 4 = PD signal present for BUSES) [7,14].

There were additional differences in the classification of detected sonographic abnormalities among the scoring systems, for example: while all scoring systems included detection of enthesophytes, certain scoring systems included this as a composite category combined with calcifications [7,19], while others made a clear distinction between the two categories and assigned different weights to both (0 or 1 for calcifications, 0–3 scale for enthesophytes) [8]. Table 1 presents an overview of the published scoring systems for enthesitis.

## 4. Discussion

Technological advances in medical imaging have led to increased applications in diagnostic evaluations, such as in the use of ultrasound to detect enthesitis. At the time of review by D’Agostino and Terslev in 2016, four major ultrasound scoring systems for enthesitis were reported: the GUESS, SEI, MASEI, and D’Agostino scoring systems [11]. In this literature review, 10 additional published ultrasound scoring systems are identified. Comparisons between these scoring systems reflect key differences in approaches to the sonographic evaluation of enthesitis around the world. Furthermore, there have also been reports on the identification of what have previously been considered to be fundamental sonographic features of enthesitis among the healthy population. Reconciling these differences and adjusting an ultrasound scoring system to address these recent findings would improve the cross-study comparison of sonographic findings and potentially improve the distinction of inflammatory from non-inflammatory causes of entheseal abnormalities detected on ultrasound.

Review of the elements of enthesitis integrated into ultrasound indices over time demonstrates the evolution in understanding of pathophysiology and sonographic detection of enthesitis. In 1999, PD was heralded as a new sonographic technique that held much promise in the evaluation of small blood vessels, but was not yet commonly used in musculoskeletal applications [23]. In order to establish the fundamental basis for the use of ultrasound in musculoskeletal imaging, Balint and Sturrock first demonstrated the low intra-observer error and improved inter-observer reproducibility of sonographic measurements of lower extremity ligaments that could be achieved [24]. The seminal work by Balint et al. in the application of ultrasound to the evaluation of enthesitis then led to the development of the GUESS scoring system in 2002, which primarily focused on structural abnormalities as could be reliably detected at the time, including tendon or ligament thickness, the absence or presence of enthesophytes or erosions, and enlargement with compressibility of associated bursae [2]. As part of this ground-breaking work, Balint et al. also consolidated key measurements of normal tendon thickness of the lower extremities and established an approach toward setting thresholds for determining abnormal tendon thickening (i.e., 0.1 mm above the reported standard deviation of each [entheseal] site in the normal population), which have since been widely used and referenced throughout the published literature since that time [4,7,8,10,13,20].

Thereafter, multiple modifications and additions to the GUESS were made, beginning with the SEI in 2007, where an attempt was made to distinguish the sonographic features of acute versus chronic entheseal injury [13]. Once again, the sonographic findings used in this scoring system focused on gray scale changes of the lower extremities and stratified the most frequent findings as tendon hypoechogenicity and increased thickness in acute enthesitis (43% and 38% of acute lesions, respectively) and bone erosion and entheseal calcification in chronic lesions (55 and 43% of chronic lesions, respectively). Interestingly, tears of the entheses were also examined as signs of chronic enthesitis in this study, but were detected in less than 10% of cases [13].

The MASEI followed thereafter in 2009, incorporating reports on the clinical utility of combining gray scale changes on B mode with vascularization identified on PD to increase sonographic detection of enthesitis, particularly in spondyloarthropathy patients [7,25]. Additionally, increased emphasis was placed on the significance of PD abnormalities in the MASEI, which utilized a weighted binary score for PD signal at the enthesis (binary score, 0 = absent, 3 = present). This study included an additional upper extremity enthesis, the triceps tendon insertion, to the previously included lower extremity entheses.

Over time, the “enthesis organ” concept by Benjamin and McGonagle, which heavily emphasized the “synovio-entheseal complex,” was becoming more widely recognized [26]. Following this concept, another modified form of the GUESS was introduced, which incorporated evaluation of the bursa along with traditional sonographic findings of enthesitis into an “inflammation score” [10]. This was in contrast to other groups who made a distinction between enthesitis and bursitis, as in the D’Agostino Scoring System, which specifically excluded isolated abnormalities of the bursa [3]. Several other European groups, including Milutinovic et al. in Serbia, identified the tendon body and associated bursa as peri-entheseal features and did not include them in the BUSES scoring system [14]. These peri-entheseal features were also excluded from the ultrasound scoring system developed by Michelsen et al. in Norway; this system adhered to a structural definition of the enthesis in the combined ultrasound evaluation for enthesitis, synovitis, and tenosynovitis to define sonographic remission in psoriatic arthritis [18].

**Figure 2:**
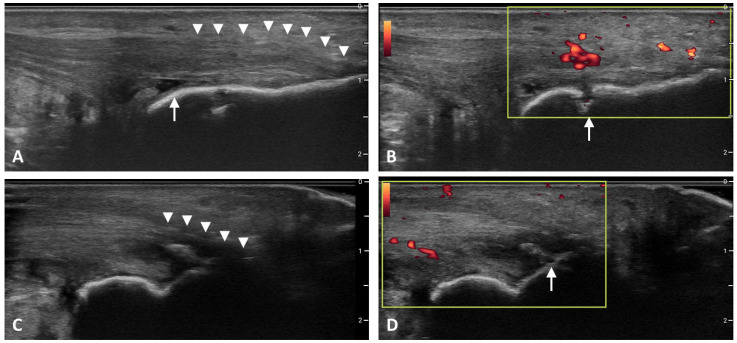
Ultrasound Findings in Enthesitis. (**A**). Entheseal thickening (arrowheads) and retrocalcaneal bursitis (arrow). (**B**). Power Doppler signal adjacent to entheseal insertion and erosion (arrow). (**C**). Hypoechogenicity (arrowheads). (**D**). Enthesophyte/calcification. Images of Achilles enthesitis in gray scale and power Doppler.

In 2018, Balint and D’Agostino collaborated through the OMERACT Ultrasound Task Force to develop a consensus-based definition of enthesitis as detected by ultrasound. In this endeavor, bursitis and tendinitis as detected by PD were discussed but ultimately believed to be separate findings that were not considered sine qua non for the detection of enthesitis, but rather as secondary findings that may be observed when inflammation has extended beyond the anatomic enthesis [6]. In addition to providing a framework for the development of an ultrasound definition for enthesitis, the OMERACT Task Force highlighted the need for future diagnostic ultrasound indices to utilize a weighted score for the severity of detected gray scale and PD abnormalities [6]. Characteristic sonographic features of enthesitis are shown in Figure 2.

While the OMERACT consensus definition on sonographic findings in enthesitis has provided a degree of standardization, multiple different approaches have continued to be used in the evaluation of enthesitis, which continues to lead to variations in clinical practice and interpretation of sonographic findings. In a study by Pukšić et al., weights were attributed to calcifications, enthesophytes, hypoechogenicity, PD signal abnormalities, and erosions at 14 entheseal sites. Notably, this scoring system was not limited to the entheses, and also included assessment of 48 joint and tendon sites for the presence of synovitis or tenosynovitis and four bursae (bilateral retrocalcaneal and deep infrapatellar) for the presence of bursitis. Composite gray scale and PD scores were calculated from summing the scores for enthesitis, synovitis, tenosynovitis, and bursitis [20]. While comprehensive, the extensive number of structures requiring ultrasound evaluation in this scoring system may have practical limitations in application to routine clinical practice. This is a similar issue faced by the scoring systems reported by Graceffa et al. and Bolkan Günaydın et al., which both require examination of 16 entheseal sites, presenting a potential barrier to application in time-limited clinical settings, and include entheses that have not been as rigorously evaluated (medial collateral ligament, greater trochanter/gluteus medius tendon, supraspinatus tendon) [19,21].

As shown in Table 1, the entheseal sites included in ultrasound scoring systems have been variable. In a systematic review, Mascarenhas and Couette found that an average of 12.7 entheseal sites are included in the ultrasound evaluation for enthesitis [12]. One concern with having a scoring index with a large number of entheseal sites is that the amount of time required to perform such extensive ultrasound evaluations may potentially limit practical applications. With this in mind, Tom et al. proposed a preliminary GRAPPA scoring system aimed at identifying the key entheseal sites that may better demonstrate inflammatory findings. Through regression modeling, six entheseal sites examined bilaterally (patellar ligament insertions into the patellar apex and tibial tuberosity, Achilles tendon and plantar fascia insertions into the calcaneus, common extensor tendon insertion into the lateral epicondyle, and supraspinatus tendon insertion into the superior facet of the humerus) were found to differentiate psoriatic arthritis patients from controls [13].

Recent studies also suggest that sonographic abnormalities of the entheses may be affected by factors other than entheseal inflammation. For example, Ben Abdelghani et al. used a binary ultrasound scoring system with simple summation to assess enthesitis among patients with primary Sjogren’s syndrome and controls. While no statistically significant differences in ultrasound detected enthesitis between these two groups were found, similar positive correlations were identified between the total number of predominantly structural entheseal abnormalities detected by ultrasound and older age (r = 0.58, *p* = 0.02 for primary Sjogren’s, r = 0.57, *p* = 0.03 for controls) [22]. Of the 250 control entheses examined, nearly a quarter (24.8%) had enthesophytes. However, a minimal fraction of these controls was noted to have increased entheseal thickness (3.2%), hypoechogenicity (1.6%), or abnormal PD signal (0.4%), suggesting that these sonographic features may better help distinguish between inflammatory and non-inflammatory entheseal pathologies compared to the findings of enthesophytes.

Other studies have demonstrated specific sonographic characteristics which are more prevalent in patients with active inflammatory conditions. These include increased tendon thickness and PD signal, which coincide with the findings reported by Ben Abdelghani et al. [12,22,27]. Similarly, Ahmed et al. found a high degree of correlation between Achilles tendon thickness and clinically active psoriatic arthritis activity based on the Psoriatic Arthritis Disease Activity Score (PASDAS) (r = 0.796, *p* < 0.001) [27]. In a prospective cohort study involving sonographic examination of 18 upper and lower extremity entheses of 111 consecutive ankylosing spondylitis patients, Wink et al. found that, among the 79.3% of patients with inflammatory features of enthesitis, most (78.4%) had increased PD signal in at least one of the entheseal sites examined, including 31.8% with involvement of the patellar ligament origin, 28.4% of the quadriceps tendon insertion, and 26.1% of the common extensor tendon origin at the lateral epicondyle [28]. D’Agostino et al. also reported high prevalence of sonographic abnormal vascularization of the entheses in spondyloarthropathy patients (detected in 81% of sonographic entheseal abnormalities), predominantly in the lower extremities [25].

Recent reports also indicate that sonographic features typically associated with enthesitis are present with variable prevalence among patients who do not have enthesitis. Based on the OMERACT consensus-based definition for enthesitis, Di Matteo et al. identified sonographic findings consistent with this definition in over 34.1% of healthy volunteers, with associated findings of entheseal thickening (28.0%), hypoechogenicity (13.4%), and PD signal (9.8%), clearly underscoring the need to further develop an ultrasound scoring system that can better distinguish enthesitis from non-inflammatory or degenerative entheseal changes [15].

One possible approach to improving this distinction could involve establishing a minimum score in an enthesitis index to minimize the inclusion of patients with degenerative changes. Based on this review of the design of previously published enthesitis indices, a framework which would include a minimum threshold to denote inflammatory findings could also be adopted, and the feasibility and validity of such a scoring system would need to be tested and validated in separate patient samples for clinical application. This new data-based index would rely on a weighted scoring system placing less emphasis on sonographic features found in otherwise healthy patients and greater emphasis on features more uniquely seen in patients with spondyloarthropathies.

Additional considerations in developing ultrasound scoring indices for enthesitis include accounting for patient-related factors that may affect findings. Bakirci et al. reported increased prevalence of sonographic features of enthesitis in individuals without rheumatologic conditions, particularly among those >50 years of age, with associated factors including male sex, older age, and high physical activity, suggesting the need to adjust for these demographic factors when designing a scoring index [16]. Specifically, Bakirci et al. found high proportions of the otherwise healthy population exhibited sonographic features of tendon thickening (86.3%) and enthesophytes (87.5%), and a low proportion exhibited findings of bone erosions (6.3%) [16]. Based on this, both enthesophytes and entheseal thickening may not be considered independently sufficient criteria for enthesitis, and lower weight could be given to the presence of these features. Similarly, as previously noted, as enthesophytes would be difficult to distinguish from calcifications close to the bone surface based on the OMERACT consensus definition for entheseal calcifications as <2 mm from the cortical bone, these could be classified as a single composite category (enthesophytes/calcifications) [6]. Given the low prevalence of erosions in the general healthy population, greater weight could be placed on their presence if detected based on a quantitative measure as previously described [10].

The increased use of ultrasound in clinical practice has opened the doors to new applications, particularly in musculoskeletal evaluation and the detection of enthesitis. However, it is important to recognize that increased detection of imaging abnormalities of the entheses may not always signify inflammatory etiologies. As new data are reported on the prevalence of sonographic changes of the entheses in the general population, ongoing modifications to existing ultrasound scoring systems may be needed to more reliably distinguish between inflammatory and non-inflammatory causes of enthesopathy.

This review provides an update on the landscape of ultrasound scoring systems and indices for enthesitis and insight into the fundamental factors to be considered in the development of an ultrasound scoring system that may reliably distinguish inflammatory from non-inflammatory changes of the entheses. Future ultrasound scoring systems will need to be implemented and applied to study samples to assess feasibility and clinical utility, as well as to validation samples to assess reproducibility. Attempts were made to minimize bias during the performance of this literature review by performing the review independently and corroborating findings following completion of the literature search with previously published reviews. However, all forms of bias may not be completely eliminated.

## 5. Conclusions

With the increasing use of ultrasound as an imaging modality for detection of enthesitis and the development of varied enthesitis scoring systems, it is important to recognize that detected sonographic abnormalities may be due to non-inflammatory causes. The published ultrasound enthesitis scoring systems are reviewed and recent findings on the prevalence of non-inflammatory enthesopathic changes are incorporated to inform the development of an ultrasound scoring system that improves the distinction of inflammatory vs. non-inflammatory causes of enthesopathy.

## Figures and Tables

**Table 1:** Published Ultrasound Scores for Enthesitis.

Study	Up Ext	Low Ext	Sites	Hypo- echo	Enthes- Ophytes	Calc	Eros	Burs	PD	Binary or Weighted Score	Ref.
GUESS Balint et al. (2002)	None	AT PF PL (P, D) QT	10	No	Yes	No	Yes	Yes	No	Binary	[2]
D’Agostino et al. (2011)	CETLA CFTME	AT GT PF PL (P) QT	14	Yes	Yes	Yes	Yes	No	Yes	Weighted	[3]
ULISSE Macchioni et al. (2019)	CETLA	AT MCL PF PL (D) QT	12	Yes	Yes	Yes	Yes	Yes	Yes	Weighted	[4]
OMERACT Balint et al. (2018)	CETLA	AT PL (P) QT	8	Yes	Yes	Yes	Yes	No	Yes	Binary	[6]
MASEI de Miguel et al. (2009)	TT	AT PF PL (P, D) QT	12	Yes	Yes	Yes	Yes	Yes	Yes	Weighted	[7]
GRAPPA (Preliminary) Tom et al. (2019)	CETLA SS	AT PF PL (P, D)	12	Yes	Yes	No	Yes	No	Yes	Weighted	[8]
Modified GUESS Aydin et al. (2013)	None	AT PF PL (P, D) QT	10	Yes	Yes	Yes	Yes	Yes	Yes	Weighted	[10]
SEI Alcalde et al. (2007)	None	AT PF PL (P, D) QT	10	Yes	Yes	Yes	Yes	Yes	No	Binary	[13]
BUSES Milutinovic et al. (2015)	CETLA	AT PF PL (P, D) QT	12	Yes	Yes	No	Yes	No	Yes	Weighted	[14]
Michelsen et al. (2016)	None (Optional)	AT PF PL (P, D) QT	10	Yes	Yes	Yes	Yes	No	Yes	Binary	[18]
Graceffa et al. (2019)	CETLA TT	AT MCL PF PL (P, D) QT	16	No	Yes	Yes	Yes	Yes	Yes	Weighted	[19]
Pukšic et al. (2018)	CETLA TT	AT PF PL (P, D) QT	14	Yes	Yes	Yes	Yes	Yes	Yes	Weighted	[20]
Bolkan Günaydin et al. (2020)	CETLA CFTME SS	AT GT PF PL (P) QT	16	Yes	Yes	Yes	Yes	Yes	Yes	Binary	[21]
Ben Abdelghani et al. (2020)	TT	AT PL (P, D) QT	10	Yes	Yes	Yes	Yes	No	Yes	Binary	[22]

Abbreviations: Up Ext = upper extremity, Low Ext = lower extremity, Sites = number of examined sites, Hypo-echo = hypoechogenicity, Calc = calcifications, Eros = erosions, Burs = bursitis, PD = power Doppler, Ref = reference, P = proximal, D = distal; Specified Sites: AT = Achilles tendon, CETLA = common extensor tendon of lateral epicondyle, CFTME = common flexor tendon of medial epicondyle, GT = greater trochanter, MCL = medial collateral ligament of knee, PF = plantar fascia, PL = patellar ligament, QT = quadriceps tendon, SS = supraspinatus tendon, TP = tibialis posterior tendon, TT = triceps tendon; Named Scoring Systems: BUSES = Belgrade Ultrasound Enthesitis Score, GRAPPA = Group for Assessment of Psoriasis and Psoriatic Arthritis, GUESS = Glasgow Ultrasound Enthesitis Scoring System, MASEI = Madrid Sonography Enthesitis Index, OMERACT = Outcome Measures in Rheumatology, SEI = Spanish Enthesitis Index. (Note: ULISSE not separately defined in original reference).

## Data Availability

The data presented in this study are available within the article and in Table 1.
